# Next‐Generation Nanopore Sensors Based on Conductive Pulse Sensing for Enhanced Detection of Nanoparticles

**DOI:** 10.1002/smll.202305186

**Published:** 2023-08-30

**Authors:** Samuel Confederat, Seungheon Lee, Der Vang, Dimitrios Soulias, Fabio Marcuccio, Timotheus I. Peace, Martin Andrew Edwards, Pietro Strobbia, Devleena Samanta, Christoph Wälti, Paolo Actis

**Affiliations:** ^1^ Bragg Centre for Materials Research University of Leeds LS2 9JT Leeds UK; ^2^ School of Electronic and Electrical Engineering and Pollard Institute University of Leeds LS2 9JT Leeds UK; ^3^ Department of Chemistry The University of Texas at Austin Austin TX 78712 USA; ^4^ Department of Chemistry University of Cincinnati Cincinnati OH 45221 USA; ^5^ Physical and Theoretical Chemistry Laboratory Department of Chemistry University of Oxford OX1 3QZ Oxford UK; ^6^ Faculty of Medicine Imperial College London SW7 2AZ London UK; ^7^ School of Molecular and Cellular Biology and Astbury Centre for Structural Molecular Biology University of Leeds LS2 9JT Leeds UK; ^8^ Department of Chemistry and Biochemistry University of Arkansas Fayetteville AR 72701 USA

**Keywords:** functionalized proteins, nanoparticle characterization, nanoparticle mixtures, nanopores, single‐molecule analysis

## Abstract

Nanopore sensing has been successfully used to characterize biological molecules with single‐molecule resolution based on the resistive pulse sensing approach. However, its use in nanoparticle characterization has been constrained by the need to tailor the nanopore aperture size to the size of the analyte, precluding the analysis of heterogeneous samples. Additionally, nanopore sensors often require the use of high salt concentrations to improve the signal‐to‐noise ratio, which further limits their ability to study a wide range of nanoparticles that are unstable at high ionic strength. Here, a new paradigm in nanopore research that takes advantage of a polymer electrolyte system to comprise a conductive pulse sensing approach is presented. A finite element model is developed to explain the conductive pulse signals observed and compare these results with experiments. This system enables the analytical characterization of heterogeneous nanoparticle mixtures at low ionic strength . Furthermore, the wide applicability of the method is demonstrated by characterizing metallic nanospheres of varied sizes, plasmonic nanostars with various degrees of branching, and protein‐based spherical nucleic acids with different oligonucleotide loadings. This system will complement the toolbox of nanomaterials characterization techniques to enable real‐time optimization workflow for engineering a wide range of nanomaterials.

## Graphical abstract

A new paradigm in nanopore research that takes advantage of a polymer electrolyte system and a conductive pulse sensing approach is presented. This system enables the analytical characterization of heterogeneous nanoparticle mixtures at low ionic strength. The wide applicability of the method is demonstrated by characterizing nanospheres of varied sizes, plasmonic nanostars, and protein‐based spherical nucleic acids.

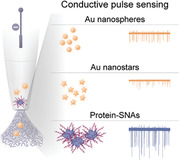

## 1 Introduction

Over the past few decades, the use of nanoparticles has played a significant role in the advancement of medicine, optics, and electronics.^[^ ^1^, ^2^, ^3^ ^]^ The use of nanoparticles not only sparked a strong engagement in the research settings, but they have also become widely incorporated in numerous consumer goods nowadays.^[^ ^4^ ^]^ Understanding the structural–functional relationship of engineered nanoparticles is a continuous undertaking that requires an in‐depth exploration of their physicochemical properties. Therefore, the ability to characterize nanoparticles in a high‐throughput manner is of utmost importance. However, characterizing nanoparticles in their native state, specifically in heterogeneous mixtures, presents many challenges.^[^ ^5^ ^]^ Dynamic light scattering (DLS) or UV–vis spectroscopy are ensemble‐averaging techniques and, therefore, fall short in fully characterizing heterogeneous nanoparticle mixtures.^[^ ^6^ ^]^ Nanoparticle tracking analysis (NTA) is suitable for analyzing the size distribution of polydisperse nanoparticle suspensions with single entity resolution; however, the nanoparticles need to have a refractive index distinct from the surrounding medium or a fluorescent label is required.^[^ ^7^ ^]^ Imaging methods such as transmission electron microscopy (TEM) provide high‐resolution characterization of individual nanoparticles but suffer from sampling bias and low throughput, typically entail *ex situ* analysis, and require careful sample preparation.^[^ ^8^ ^]^

Nanopore sensing is a powerful label‐free electrical technique that uses the Coulter principle for single‐entity analysis.^[^ ^5^ ^]^ In nanopore experiments, individual entities are driven through a nanopore under the influence of an electric field, causing a temporary modulation in the recorded ion current by a combination of geometrical exclusion of the electrolyte solution, ion concentration polarization, and additional charges brought by the analyte itself.^[^ ^9^, ^10^ ^]^ The magnitude and duration of these modulations reflect the translocation dynamics of the analyte, which are dependent on its physicochemical properties (e.g., size, shape, charge).^[^ ^11^, ^12^, ^13^ ^]^ Even though nanopores have been employed in numerous sensing applications, nanopore technology has an untapped potential for the analysis of nanoparticles.^[^ ^14^ ^]^ This is because current nanopore measurements require the size of the pore to match the size of the analyte, limiting the investigation to homogenous mixtures.^[^ ^15^, ^16^, ^17^ ^]^ Furthermore, nanopore measurements often require high‐ionic‐strength electrolytes which precludes the analysis of nanoparticles systems that are unstable at high ionic strength.^[^ ^18^, ^19^ ^]^ Tuneable resistive pulse sensing (TRPS) is a nanopore technique that has found applications in nanoparticle characterization, but it is limited to nanoparticles larger than 100 nm in size.^[^ ^20^, ^21^ ^]^ Furthermore, the TRPS signals deviate from linearity when the size of the nanoparticle approaches the diameter of the pore aperture, limiting its analytical capabilities.

Here, we present a polymer‐electrolyte‐enhanced conductive‐pulse nanopore sensing approach which enables the analysis of heterogeneous nanoparticle samples at low ionic strength. The polymer electrolyte environment generates a large signal enhancement eliminating the need for a nanopore that matches the size of the nanoparticle and therefore allowing the high throughput analysis of heterogeneous nanoparticle mixtures. Furthermore, combining experimental findings and finite‐element modelling (FEM), we provide a mechanistic explanation for the ion current signatures. We demonstrate the characterization of nanoparticles at low ionic strength (10 mm KCl) enabling the analysis of anisotropic gold nanostars (AuNS) with varying degrees of branching and report the detection and analysis of an emerging class of functional soft nanoparticles, protein spherical nucleic acids (ProSNAs) with distinct oligonucleotides shells. The single‐nanoparticle analysis approach described herein will complement the toolbox of existing nanomaterials characterization techniques, unleashing the potential of nanopore sensing for the universal analysis of nanoparticles.

## 2 Results and Discussion

**Figure 1:**
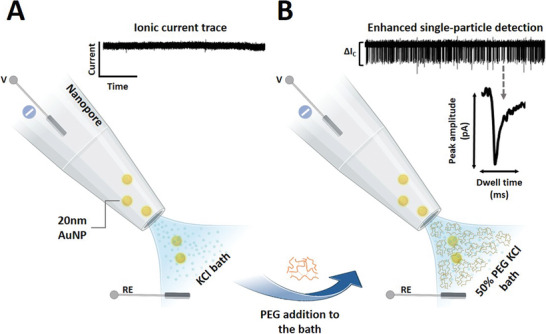
Enhanced nanoparticle detection in a polymer electrolyte bath. A) Schematic illustration depicting 20 nm diameter AuNPs translocating across the nanopore into the outer bath and the recorded ion current trace at −500 mV for 20 nm AuNPs utilizing a 50 mm KCl external electrolyte bath. No translocation events were observed under these conditions. B) Representative translocation setup depicting the use of 50% (w/v) PEG (polymer electrolyte) in 50 mm KCl in the external electrolyte bath with the corresponding recorded ion current trace at −500 mV, showing conductive translocation events for 20 nm AuNPs. A representative translocation event is shown with the translocation peak amplitude and dwell time characteristics indicated.

A polymer electrolyte nanopore system^[^ ^22^ ^]^ enables the detection of heterogeneous nanoparticle mixtures with a fixed nanopore size.^[^ ^23^ ^]^ We fabricated glass nanopores with a diameter of 60 nm and probed the translocation of 20 nm diameter gold spherical nanoparticles (AuNPs) samples (**Figure** **1**) under an applied voltage of −500 mV. The nanopore setup consisted of a glass nanopore filled with and immersed in an electrolyte (KCl) solution where the application of a potential between a pair of Ag/AgCl electrodes, inside the glass nanopore and external bath drives the translocation of the analyte toward the external bath. As shown in Figure 1A, no translocation events were observed for the 20 nm AuNPs using standard electrolyte buffer conditions (i.e., 50 mm KCl solution). However, the addition of 50% w/v PEG (polymer electrolyte) to the outer bath resulted in conductive translocation events well‐resolved from the ion current baseline (Figure 1B). These events are characterized by a peak amplitude (Δ*I*_c_) and dwell time (Figure 1B inset), where each peak is generated by individual nanoparticles translocating from inside the pore to the outer bath. No events were observed under the application of a positive voltage or in absence of nanoparticles in solution (Figure S1, Supporting Information). Increasing the magnitude of the applied voltage led to an increase in amplitude of the conductive peak current and a decrease in the dwell time, suggesting that the electrophoretic force has a major contribution in driving the negatively charged AuNPs through the pore (Figure S2, Supporting Information). Similarly, we observed a linear increase in the frequency of the translocation events with increasing voltage and increasing nanoparticle concentrations (Figure S2, Supporting Information).

**Figure 2:**
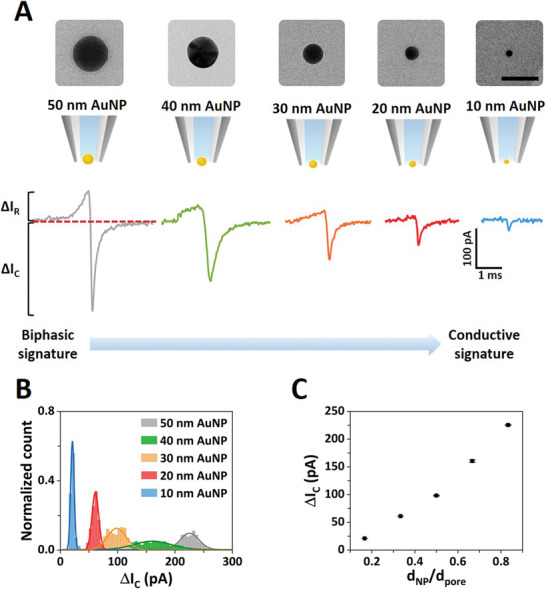
Nanopore detection of gold nanoparticle mixtures. A) Top panel: TEM images of the standard AuNPs, left‐to‐right: 50, 40, 30, 20, and 10 nm diameter (50 nm scale bar). Bottom panel: schematic representation of the AuNP translocating through a 60 nm diameter fixed pore size with representative individual translocation peaks for each AuNP, denoting a transition from biphasic to conductive signature when the ratio of nanoparticle to pore size decreases. B) Event histogram of the conductive peak current for the five different AuNPs (50, 40, 30, 20, and 10 nm diameter) translocated with a fixed pore size (≈60 nm diameter) under a ‐500 mV applied voltage. The solid lines represent Gaussian fits to each translocation data set. The distribution of the 40 nm sample is wider because of a 15% coefficient of variation of the nanoparticle size compared to 5% for the other samples (see Experimental Section for further details). C) Average conductive peak current as a function of *d*_NP_/*d*_pore_. The error bars represent the standard error of the measurements presented in part (B). Comparable measurements made with different ≈60 nm diameter pores are shown in Figures S4 and S5 (Supporting Information).

Figure S3 (Supporting Information) depicts the translocation of a mixture of 50 nm and 20 nm diameter AuNPs where two distinct peak amplitudes can be observed that also result in distinct populations in the events scatter plots (Figure S3C, Supporting Information). The average peak amplitude was 61.1 ± 1 pA for the 20 nm AuNPs and 225 ± 1 pA for the 50 nm AuNPs. The large amplitude difference denotes the strong signal enhancement generated by the polymer electrolyte that allowed us to further probe a range of different AuNPs (50, 40, 30, 20, and 10 nm diameter) with a fixed nanopore size (60 nm in diameter) as shown in **Figure** **2**A. In contrast to previous nanopore strategies employing chemical modifications^[^ ^24^ ^]^ or arrayed nanopores,^[^ ^25^ ^]^ our nanopore system supports rapid detection of heterogeneous nanoparticles and clear discrimination of their diameter with a fixed pore diameter. Whereas previous studies demonstrated the discrimination of nanoparticle mixtures utilizing a single nanopore, they were predominantly used for the analysis of binary mixtures with relatively large difference in their size.^[^ ^26^, ^27^ ^]^ Here, we further explored the discrimination of nanoparticles with 10 nm size difference in the low nanometer range (10–50 nm). The translocation signal distributions were fitted to Gaussian curves which enabled the identification of 5 distinct populations, one for each nanoparticle set (Figure 2B). The average conductive peak current increased with the ratio of the nanoparticle diameter to the pore diameter (*d*_NP_/*d*_pore_), as shown in Figure 2C. To further demonstrate the capability of our nanopore approach to discriminate heterogeneous nanoparticles mixtures, in Figure S4 (Supporting Information) we compared the translocation of individual AuNP solutions with a mixture containing all AuNPs (50, 40, 30, 20, and 10 nm) in one solution.

**Figure 3:**
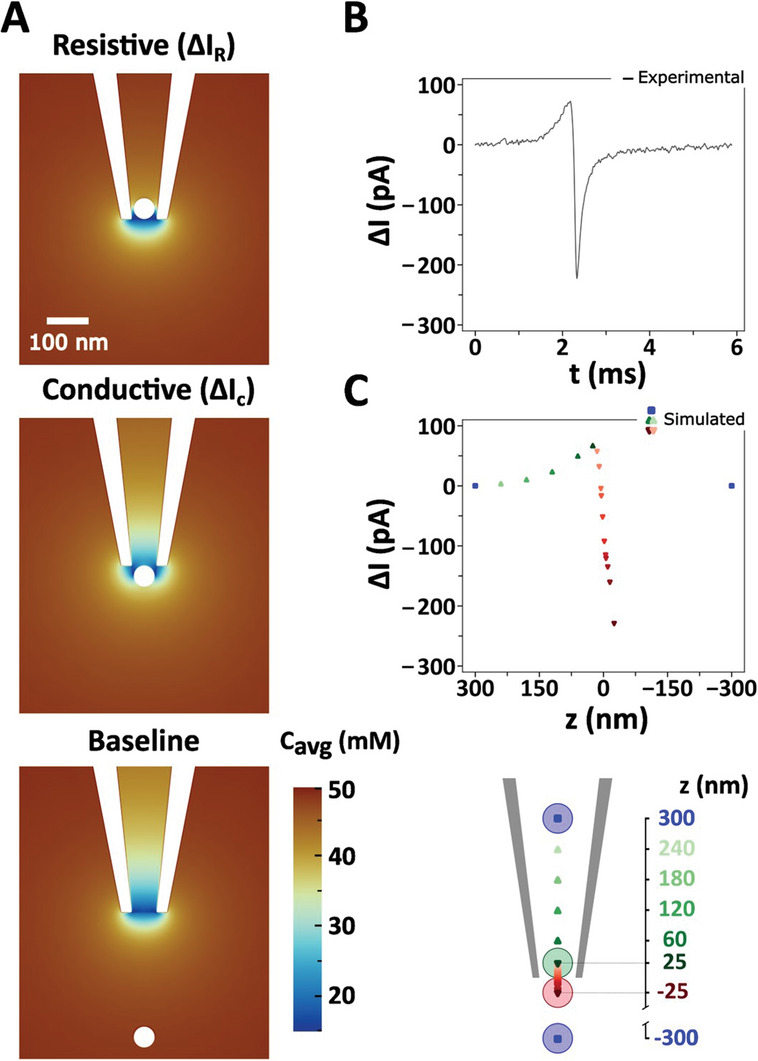
Finite element simulations of AuNP translocation. A) Simulated ion distribution in the proximity of the nanopore tip utilizing a 50% PEG in 50 mm KCl electrolyte bath with a 50 nm diameter AuNP at different translocation positions through a 60 nm diameter pore (top: inside the nanopore, middle: at the nanopore tip, bottom: outside in the bath) under a −500 mV applied voltage. B) Experimental and C) simulated translocation signal of the 50 nm diameter AuNP through a 60 nm diameter pore with 50% PEG in 50 mm KCl electrolyte bath, and a nanopore schematic depicting the translocation distance used for simulated translocation signal (bottom panel).

Whereas the conductive peak represents the dominant feature in the ion current signatures, we observed a distinct change in the translocation signature when different nanoparticle to pore size ratios were employed. Namely, when the size of the nanoparticle closely matched the size of the nanopore, an additional resistive peak was observed (Figure S5, Supporting Information). This behavior is evidenced in Figure 2A, where a biphasic (resistive and conductive peak) signal transitions into a conductive signal when the size of the AuNP decreases with respect to the size of the pore (Table S1, Supporting Information) or vice versa (Figure S6, Supporting Information). Classically, nanoparticle analysis with nanopores leads to resistive (current‐decreasing) translocation signals because of the ion flow hindrance by the presence of the nanoparticle within the nanopore sensing region.^[^ ^26^ ^]^ However, several studies have shown the occurrence of conductive peaks. Sensale et al. identified the surface charge of the particle as the main factor that influences the characteristics of the translocation signal through a glass nanopore.^[^ ^28^ ^]^ The authors suggested that this phenomenon occurs due to an ion accumulation/depletion associated with the surface charge of the particle translocating through the pore. Similarly, in the studies conducted by Menestrina et al. and Chen et al., biphasic signals were reported both experimentally and in simulations when charged particles are translocated in low salt buffer conditions (below 200 mm KCl).^[^ ^29^, ^30^ ^]^ The authors reported resistive peaks followed by a conductive component and explained the effect considering the charge carried by the analyte that causes a temporary ion enrichment at the aperture when low‐salt conditions are used. The phenomenon of the current enhancement can be influenced by several factors, including the electrolyte concentration, nanoparticle to pore size ratio, and the nanoparticle surface charge.^[^ ^31^ ^]^ To investigate the origin of the conductive contribution to the peak in our nanopore system, we developed a finite‐element model to estimate the resulting current enhancement when a polymer electrolyte (50% PEG) is used in the external solution. We have recently shown that the polymer electrolyte induces a polarity‐dependent ion distribution and transport at the nanopipette tip region. We also demonstrated that a combination of the unique ion transport behavior and the interaction between a translocating molecule and the polymer electrolyte interface is responsible for the increased magnitude in the translocation signals.^[^ ^32^ ^]^ Here, we hypothesized that an interface is formed at the nanopore between the inner and outer solution (KCl only and the polymer electrolyte bath), and that this interface is deformed by a translocating spherical nanoparticle (50 nm diameter). This deformation induces an ionic rearrangement within the nanopore producing the single nanoparticle events enhancement as we observed before with the translocations of nucleic acids.^[^ ^32^ ^]^ The resistive component in **Figure** **3**B is due to the “classic” volume exclusion effect of resistive pulse sensing which strongly correlates with the nanoparticle to nanopore size ratio (Figure 2A). Interestingly and counter‐intuitively, as the nanoparticle travels through the nanopore sensing area an increase in the ionic concentration is observed due to the deformation of the PEG interface. This interface deformation is responsible for the presence of the conductive peak. To further highlight the influence of the interface on the conductive peak enhancement, we simulated the effect of a negatively charged spherical nanoparticle versus a neutral nanoparticle of similar size. As shown in Figure S7 (Supporting Information), the magnitude of the conductive peak current is largely influenced by the external polymer electrolyte interface, with small contribution exerted by the particle surface charge. Remarkably, although we simulated the effect of the presence of a nanoparticle at distinct locations within the nanopore at equilibrium for each position (Figure 3A), we observed an excellent agreement with the experimentally measured resistive and conductive signal (Figures 3B,C). We note that we do not have experimental data on the nature of the interface at the nanopore between the inner and the outer bath and therefore we assumed it to be a straight boundary at *z* = 0 nm. This is clearly an over‐simplification, and the interface geometry is likely to be more complex. Also, our stationary and ergodic model may not capture the full dynamics of the nanoparticle translocation across the interface, but it does capture the underlying process and provides a good phenomenological understanding of the impact of the nanoparticle translocation on the ion current. Using a more sophisticated fully dynamic model would certainly improve the accuracy of the predictions, but would unlikely lead to a significant revision of the phenomenological understanding of the system.

**Figure 4:**
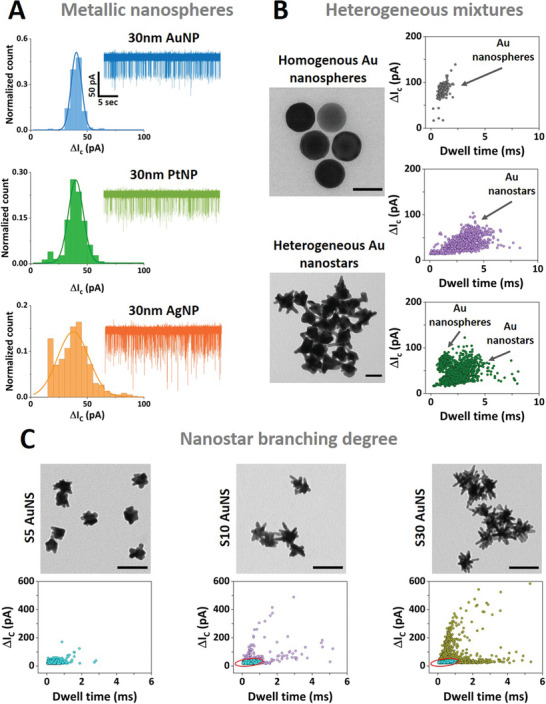
A) Nanopore sensing of metallic nanospheres with histograms of the conductive peak current distribution and representative ion current traces for: 30 nm diameter PEG carboxyl‐capped gold nanospheres (top panel), 30 nm diameter citrate‐capped platinum nanospheres (middle panel), and 30 nm diameter citrate‐capped silver nanospheres (bottom panel). The solid lines represent Gaussian fits to each translocation data set. Nanopore recordings were carried out in 50% PEG in 10 mm KCl utilizing a 60 nm diameter fixed pore size biased at −500 mV. The current and time scales are the same for all the ion current traces. B) Nanopore sensing of homogenous gold nanospheres (TEM top panel) and heterogeneous gold nanostars (TEM bottom panel). Scale bar TEM graphs: 50 nm. Scatter plots with conductive peak current (Δ*I*_C_) versus dwell time of the translocation events are depicted for the individual sets (top panel: gold nanospheres; middle panel: gold nanostars), and a mixture (bottom panel: gold nanospheres and gold nanostars) in similar nanopore conditions (50% PEG in 10 mm KCl using an 80 nm diameter fixed pore biased at −500 mV). C) Nanopore sensing of nanostars with different branching degrees of three sets of synthesized gold nanostars as depicted in the TEM images: S5 (left panel), S10 (middle panel), and S30 (right panel) AuNS (100 nm scale bar). The notation S5–S30 denotes their different branching degree (from low to high). Scatter plots of conductive peak currents as a function of dwell time for the S5 AuNS sample (left panel), S10 AuNS sample (middle panel), and S30 AuNS sample (right panel). The red ellipse in the S10 and S30 scatter plot data indicates the 95% confidence interval (CI) of the S5 translocation events. 12% of the events fall outside the S5 CI for the S10 sample and 35% of the events fall outside the S5 CI. Nanopore recordings were carried out in 50% PEG in 10 mm KCl conditions utilizing 80 nm diameter pores biased at −700 mV.

Our polymer electrolyte system also enables the analysis of nanoparticles at low ionic strength (10 mm). Generally, nanopore measurements are carried out in high‐salt conditions (>100 mm KCl), excluding the analysis of less stable nanoparticles, such as citrate‐capped nanoparticles.^[^ ^33^ ^]^ We probed the translocation of gold and platinum citrate‐capped nanoparticles and bare silver nanoparticles diluted in 10 mm KCl, demonstrating that our nanopore measurements can reliably detect nanoparticles with high‐capture rates and high signal‐to‐noise ratios (SNR). These results are evident in **Figure** **4**A, where the translocation of three sets of 30 nm metallic nanospheres (Figure S8, Supporting Information) utilizing a 60 nm nanopore biased at −500 mV. The data also shows a good agreement between the average conductive peak current recorded for each set of nanoparticles with nominally the same size (30 nm diameter). The Gaussian fits of the peak current distribution in Figure 4A resulted in a average conductive peak current of 40 ± 1 pA for the AuNPs, 40 ± 1 pA for the PtNPs, and 38 ± 1 for the AgNPs, respectively.

Inspired by the results above, we expanded our nanopore measurements to tackle complex anisotropic nanoparticles, such as branched gold nanostars (AuNS). AuNS are emerging as prominent plasmonic particles for application in surface‐enhanced Raman scattering (SERS) and offer several advantages over spherical nanoparticles.^[^ ^34^, ^35^, ^36^ ^]^ The localized surface plasmon in AuNS is tuneable by controlling the anisotropy of the structure during the synthesis.^[^ ^37^ ^]^ However, current characterization and quality control for these plasmonic nanostructures relies mainly on TEM imaging. We first probed a nanoparticle solution composed of AuNS spiked with 50 nm gold nanoparticles. Their nominal hydrodynamic radius was broadly similar (60 nm vs 50 nm), but the ion current signals generated two clearly distinct populations indicating the potential of our approach for the quantitative analysis of anisotropic nanoparticles (scatter plots Figure 4B). Opposite to the translocation events obtained for the uniform gold nanospheres, the events recorded for the AuNS samples show a wider spread in terms of the peak current and dwell time. We attribute these differences to the irregular shape of the synthesized nanoparticles and their heterogeneous character, as depicted in the TEM images in Figure 4B. Furthermore, we probed suspensions of AuNS with low and high degree of branching in 50% PEG and 10 mm KCl following their different synthesis stages (Figure 4C). Similarly, we use a fixed nanopore size (80 nm diameter) to probe the translocation of distinct AuNS samples (Figure 4C), here named S5, S10, S30, respectively (see Gold Nanostar Synthesis section), according to their degree of branching. The samples all exhibit a large amount of anisotropy which gives rise to their unique optical properties (Figures S9 and S10, Supporting Information). With increasing branching density, we observed a broadening of the distributions both in terms of peak current amplitude and dwell time as shown in the scatter plots in Figure 4C and Figure S11 (Supporting Information). To evidence the progression in terms of nanopore detection from synthesis of AuNS (S5) to the high‐density branched AuNS (S30) we computed a 95% confidence interval (CI) using the S5 translocation events as an input. We then applied this CI to the translocation events obtained for S10 AuNS and S30 AuNS samples. Based on this CI fitting we outlined an increase in the percentage (from the total number of events) of the events falling outside the S5 CI, namely 12% for the S10 and 35% for the S30 AuNS. In future, more sophisticated analysis employing algorithmic models could reveal more subtle information concerning the ion current signals.

**Figure 5:**
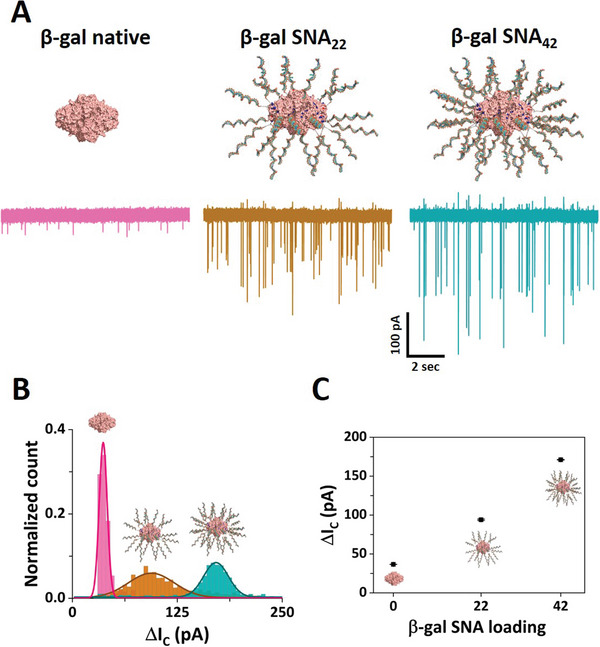
Nanopore sensing of protein spherical nucleic acids. A) Nanopore translocation ion current traces obtained for the native β‐gal (left), β‐gal SNA_22_ (middle), and β‐gal SNA_42_ (right). The current and time scales are the same of all the ion current traces. B) Histograms of the conductive peak current for native β‐gal (pink bars), β‐gal SNA_22_ (brown bars), and β‐gal SNA_42_ (teal bars). C) Average conductive peak current as a function of the oligonucleotide loading. The error bars represent the standard error. Nanopore measurements were carried in 50% PEG and 100 mm KCl using a 30 nm diameter pore size biased at −700 mV.

To further expand on the applicability of our polymer electrolyte enhanced nanopore system for investigating various functional nanostructures,^[^ ^38^, ^39^, ^40^ ^]^ ProSNAs are based on the spherical nucleic acid (SNA) architecture and consist of a protein core functionalized with a dense shell of DNA strands.^[^ ^38^ ^]^ Compared to its analogous native protein, ProSNAs, i.e., the protein functionalized with ssDNA (single‐stranded DNA), show an increase in cellular uptake, with the oligonucleotide density playing a critical role in the cellular uptake efficiency.^[^ ^40^ ^]^ Hence, a rational investigation of the DNA‐mediated functionalization of these hybrid nanostructures can support the development of new architectures. Here, we used β‐galactosidase (β‐gal) ProSNAs thanks to their well‐established stability and ease of synthesis (Figure S12, Supporting Information). As depicted in **Figure** **5**A, our polymer electrolyte enhanced sensing approach enabled the detection of the native β‐gal protein and β‐gal SNAs with two different DNA loadings: 22 ssDNAs (β‐gal SNA_22_) and 42 ssDNAs (β‐gal SNA_42_), respectively (Figures S13 and S14 and Table S2, Supporting Information). The average conductive peak current obtained for the native β‐gal protein was centered at 32 pA, while a substantial increase was observed for the β‐gal SNA samples with a peak centered at 94 pA for the β‐gal SNA_22_ and 171 pA for β ‐gal SNA_42_ (Figure 5B,C). Similarly, an increase in the translocation duration was observed between the native β‐gal protein and β‐gal SNAs (Table S3 and Figure S15, Supporting Information). Importantly, apart from the clear discrimination between the native protein and the DNA‐functionalized counterparts, we were also able to differentiate the β‐gal SNAs with two different DNA loadings (SNA_22_ and SNA_42_). Remarkably, even at low DNA loading (SNA_22_), comprising only several sparsely attached ssDNA to the protein surface, we were able to provide a clear discrimination from the bare protein serving as scaffold for attachment. In similar fashion, we showed the ability to differentiate between the two different ssDNA loadings, with a proportional increase in the peak current and dwell time with the increasing density of the ssDNAs attached to the protein. These results are in line with the ≈two‐fold increase in the DNA shell density of the functionalized protein. This rapid yet easy‐to‐implement nanopore sensing allowed us to probe small variations in the density of the soft DNA shell on the surface of the protein. Taken together, these results show that the polymer‐electrolyte nanopore system can enable the characterization of soft nanomaterials and allow for the discrimination of nanoparticles based on their functionalization state. These measurements provide a basis to investigate functionalization strategies with biomolecules (i.e., different densities/moieties) for hybrid materials at the nanoscale level with implications in cellular imaging, sensing, and drug delivery.^[^ ^41^, ^42^ ^]^

## 3 Conclusions

We have demonstrated the development and implementation of a nanopore system enhanced by a polymer electrolyte to comprise a conductive pulse sensing approach enabling the analytical characterization of nanoparticles. Using experiments and finite element modeling, we provided a mechanistic description of the ion current signals and further employed the system to analyze heterogeneous gold nanoparticle mixtures. We then demonstrated the unique ability of our approach to fingerprint nanoparticle samples at low ionic strength (10 mm) and exemplified the power of the system by characterizing the degree of branching of “hard” anisotropic Au nanostars and the nucleic acid coverage of “soft” ProSNAs. This universal system will complement the toolbox of nanomaterials characterization techniques and enable the real‐time optimization of flow synthesis of a wide range of nanomaterials.

## 4 Experimental Section

### Chemicals and Materials

All reagents used in the translocation experiments were prepared using ultra‐pure water (18.2 MΩ cm) from Millipore system and further filtered through a 0.22 µm syringe. KCl, Triton‐X, EDTA, and PEG reagents were purchased from Sigma Aldrich. UltraUniform (5% CV) Gold PEG carboxyl‐capped nanospheres (10, 20, 30, and 50 nm diameter) were purchased form NanoComposix, BioReady (15% CV) 40 nm Gold PEG carboxyl‐capped nanospheres, silver citrate‐capped nanospheres (30 nm diameter), and platinum citrate‐capped nanospheres (30 nm nanospheres) were purchased from NanoComposix. Silver wire (0.25 mm diameter) used in the nanopore experiments was obtained from Alfa Aesar.

### Standard Nanoparticle Characterization

The stability of the gold nanoparticles diluted in the KCl translocation buffer was probed by UV–vis measurements (Figure S16, Supporting Information) using a NanoDrop ND‐1000 spectrophotometer (Thermo Scientific). The size distribution of the standard nanoparticles in solution was determined by Zetasizer NanoZS (Malvern Instruments Ltd.) (Figure S16, Supporting Information). All the standard nanosphere samples were used as received.

### Nanopore Fabrication and Characterization

The nanopores were fabricated starting from 1.0 mm × 0.5 mm quartz capillaries (QF120‐90‐10; Sutter Instrument, UK) with the SU‐P2000 laser puller (World Precision Instruments, UK), using a two‐line program: 1) HEAT, 750; FILAMENT, 4, VELOCITY, 30; DELAY, 145, PULL, 80; 2) HEAT, 600, FILAMENT, 3; VELOCITY, 40; DELAY, 135; PULL, 150. The pulling parameters are instrument specific and lead to a glass nanopore with a diameter of ≈60 nm. Adjustments of the HEAT and PULL parameters were made to fabricate other pore sizes specified in this study. The pulled glass nanopores were characterized by measuring their pore resistance in 0.1 m KCl and the pore dimensions were confirmed by Scanning Electron Microscopy (SEM) using a Nova NanoSEM at an accelerating voltage of 3–5 kV. The characterization of 60 nm glass nanopore is exemplified in Figure S17 (Supporting Information).

### Polymer Electrolyte Preparation

The KCl electrolyte was first dissolved with 18.2 MΩ ddH_2_O to a final concentration of 1 m, the solution was then filtered through a 0.22 µm syringe membrane filter (E4780‐1223; Starlab UK). For example, to generate 10 mL of the 50% (w/v) PEG with 50 mm KCl, 0.5 mL of the 0.22 µm filtered 1 m salt solution, 4.5 mL of 0.22 µm filtered 18.2 MΩ ddH_2_O and 5 g of PEG 35 kDa (ultrapure grade, Sigma Aldrich) were mixed inside a tube. The tube was then left inside a 70 °C incubator for 2 h and then kept at 37 °C overnight. The tubes were then left on a lab bench for 4 h to reach room temperature prior use. The polymer electrolyte was then stored at room temperature.

### Nanopore Translocation Measurements

Unless otherwise specified, the translocation experiments were carried out by filling the glass nanopore with the translocation buffer (50 mm KCl, 0.01% Triton‐X, 10 mm Tris, 1 mm EDTA, pH 8.0) containing the nanoparticles. The glass nanopore was then immersed in a similar buffer with the addition of 50% (w/v) Polyethylene Glycol (PEG) 35 kDa (ultrapure grade, Sigma Aldrich). The notation of the 50% PEG in the text refers to the utilization of 50% (w/v) Polyethylene Glycol (PEG) 35 kDa. A Ag/AgCl wire (0.25 mm diameter, GoodFellow UK) was inserted in the glass nanopore barrel and acted as the working electrode, while a second Ag/AgCl wire was immersed in the bath and acted as the counter and reference electrodes. The nanoparticles were driven from inside the glass nanopore into the external bath by applying a negative potential to the working electrode placed inside the glass nanopore with respect to the reference electrode in the bath. The ion current was recorded with a MultiClamp 700B patch‐clamp amplifier (Molecular Devices) in voltage‐clamp mode. Data were acquired at a 100 kHz sampling rate with a 20 kHz low‐pass filter using the pClamp10 software (Molecular Devices). The ion current traces were further analyzed with the MATLAB script Transalyzer, developed by Plesa et al.^[^ ^43^ ^]^ The obtained translocation events were analyzed by applying a 7‐sigma threshold level from the baseline, and only the events above the threshold were considered as translocation events (Figure S18, Supporting Information). The obtained events were further analyzed and plotted using Origin 2019b.

### Numerical Simulations

To provide a mechanistic understanding of the experimentally observed current responses during nanoparticle translocation in the system, numerical simulations describing the electric potential, ion concentrations, and fluid flow within and around the aperture of the glass nanopore were developed. The simulations, which are described briefly below, were based on a model developed previously to describe a glass nanopore immersed in a polymer electrolyte.^[^ ^32^ ^]^ For a more detailed description, including a full list of parameters and boundary conditions, see Figure S19 and Table S4 (Supporting Information).^[^ ^32^ ^]^ The commercial finite element software COMSOL Multiphysics (version 5.6) was used to solve the equations describing the spatially varying quantities listed above. Boundary conditions were selected to reflect the experimental system, including the surface charge on the glass/nanoparticle and the bulk solution concentrations. K^+^ and Cl^−^ transport depends on the phase (PEG+KCl or KCl) with diffusion coefficients chosen to match the experimentally measured solution conductivities. In KCl, the diffusion coefficients of the K^+^ and Cl^−^ are approximately equal^[^ ^44^ ^]^ (D_K+_:D_Cl−_ = 0.49:0.51) while in PEG this ratio becomes D_K+_:D_Cl−_ = 0.35:0.65, as previously determined.^[^ ^32^ ^]^ The latter reflects cation affinity to PEG. For simplicity, the interface between the PEG+KCl and KCl was taken to have zero width, i.e., mixing of the solutions was neglected. In the absence of a nanoparticle, the interface was taken to be the disk exactly at the mouth of the pipette, while a nanoparticle translocation was taken to perturb this interface outward (see Figure S19, Supporting Information). The current was calculated by integrating the ion flux at the top of the nanopore.

### Gold Nanostar Synthesis

First, citrate‐capped gold nanoparticle (12 nm) seeds were synthesized using the Turkevich method.^[^ ^45^ ^]^ Briefly, 100 mL ultra‐pure boiling water and 200 µL of 0.5 m HAuCl_4_ were dispersed for 10 s. 15 mL of 1% trisodium citrate was added. After the final color change, the solution was boiled for 15–30 min. Then the solution was cooled, filtered through a 0.22 µm nitrocellulose membrane, and stored at 4 °C until further use. The Au nanostar synthesis followed the Vo‐Dinh group surfactant‐free procedure with a few modifications.^[^ ^34^ ^]^ In brief, under room temperature and fast stirring, 10 mL of ultra‐pure H_2_O, 10 µL 1 n HCl, and 493 µL of 0.5 m HAuCl_4_ were added to a plastic scintillation vial. Then, 100 µL of Au seeds were added, and 10 s afterward AgNO_3_ of various concentrations (1–3 mm; samples named S5, S10, and S30 relating to the AgNO_3_ final concentration, 5, 10, and 30 µm, respectively) and 50 µL of 0.1 m L‐Ascorbic Acid were simultaneously quickly added. Finally, 10 s afterward, the stirred solution of Au nanostars was transferred and immediately used. After the Au nanostar synthesis, 325 µL of thiol stabilizer (HS‐PEG‐COOH) was added to the Au nanostars, they were vortexed, and then left idle for 30 min at room temperature. After 30 min, the solution was centrifuged and washed 3 times with ultra‐pure H_2_O (1500 g, 15 min, 4 °C). Lastly, the samples were redispersed in ultra‐pure H_2_O and stored at 4 °C until used.

### Gold Nanostar Characterization

Au nanostars with thiol stabilizer were prepared for imaging by dropping 10 µL of two‐fold diluted sample onto a 200‐mesh grid. The sample was allowed to dry overnight at room temperature. Then transmission electron microscope (TEM; Hitachi H7650) imaging was performed at 80 kV with an AMT BIOSPR16 camera (Figure S10, Supporting Information). To determine the size and concentration, the samples were measured via NanoSight NS300 nanoparticle tracking analysis (NTA) system with a 532 nm laser. 20‐, 40‐, and 80‐fold dilutions of the samples were used for the measurements. Extinction spectra of the samples were collected via a Biotek Microplate reader (Figure S9, Supporting Information).

### DNA Synthesis and Characterization

All oligonucleotides used in this work (Table S2, Supporting Information) were synthesized using a MerMade 6 instrument (LGC, Biosearch Technologies) at 1 µmol scale. Reagents for DNA synthesis were purchased from Glen Research. Controlled pore glass (CPG) beads (Glen Research, Cat. No. 20‐5041‐10) were used as the solid support for DNA synthesis. The synthesized products were cleaved from the CPG beads and deprotected using 0.5 mL of 30% ammonium hydroxide (Thermo Fisher, Cat. No. A669) for 16 h at room temperature. After 16 h, the solution was run through a NAP‐5 column (Cytiva illustra, Cat. No. 17085301) following manufacturer's protocol. The eluate was purified using reversed phase HPLC (Vanquish HPLC, Thermo Fisher, USA) using a C18 column (Thermo Fisher, Cat. No. 41005‐259070A). A gradient of 0 to 70% A→B over 40 min was used. A was 30 mm triethylammonium acetate (Thermo Fisher, Cat. No. O4885‐1) with 3% (v/v) acetonitrile (Thermo Fisher, Cat. No. A996SK‐4) and B was 100% acetonitrile. The collected fractions were lyophilized and redissolved in water. The mass of the oligonucleotides was determined using matrix‐assisted laser desorption ionization time‐of‐flight mass spectrometry (MALDI‐TOF MS, Bruker, AutoFlex Max) using 2′,6′‐dihydroxyacetophenone (DHAP, Sigma, Cat. No. 37468) as a matrix. The absorbance (*A*_λ_) of oligonucleotides was measured as a function of wavelength (λ) using UV–vis spectroscopy (Agilent, Cary 60 UV–vis Spectrophotometer) using a quartz cuvette (Thermo Fisher, Cat. No. 50‐753‐2877) with 1 cm pathlength. Using the extinction coefficient (ε_λ_) at 260 nm (obtained from the IDT Oligo Analyzer tool), the concentration (*c*) of the purified products was determined using Beer's law (Equation (1)):

(1) $$A_{\lambda }=\varepsilon_{\lambda }\;cl;\;\;l\;=\;1\;\;\mathrm{cm}$$

### ProSNA Synthesis and Characterization

Briefly, surface cysteine groups are first modified with Alexa Fluor 647 C_2_ maleimide. Following this step, NHS‐PEG_4_‐azides are conjugated to the surface‐accessible lysine residues^[^ ^38^, ^39^ ^]^, as shown in Figure S12 (Supporting Information). Finally, DBCO‐terminated DNA is attached to the azide‐modified proteins through copper‐free click chemistry. Details of the synthesis steps are described in Section S11 (Supporting Information). The synthesis of β‐gal SNAs was confirmed through UV–vis and native PAGE gel electrophoresis, as shown in Figures S13 and S14 (Supporting Information).

## Conflict of Interest

The authors declare no conflict of interest.

## Supporting information

Supporting Information

## Data Availability

The data that support the findings of this study are openly available from the University of Leeds data repository at https://doi.org/10.5518/1335.
